# Cytoprotection against Cadmium by Nrf2-Activating Dietary Phytochemicals

**DOI:** 10.24966/acim-7562/100523

**Published:** 2024-10-14

**Authors:** Brooks M Hybertson, Bifeng Gao, Swapan Bose, Joe M McCord

**Affiliations:** 1 Pathways Bioscience, Aurora, USA; 2 Department of Medicine, Division of Pulmonary Sciences and Critical Care Medicine, University of Colorado Anschutz Medical Campus, Aurora, USA

**Keywords:** Aging, Cadmium, KEAP1, Metallothionein, Nrf2

## Abstract

**Background:**

Environmental exposure to toxic heavy metals such as cadmium continues to cause significant human health impacts around the world.

**Objective:**

This aim of this study was to determine potential benefits of a Nrf2 activating phytochemical combination against cadmium-induced cytotoxicity.

**Methods:**

Human Hepatocellular Carcinoma (HepG2) cells were treated with the Nrf2-activating PB123 combination of dietary phytochemicals and the cultured HepG2 cells were challenged with cadmium (CdSO4). Cytotoxicity effects were evaluated by assays of cell viability (CCK8 assay) and cell injury (LDH release assay). Gene mRNA expression in PB123-treated cells was determined by RNA-seq.

**Results:**

PB123 upregulated metallothionein gene expression in the HepG2 cells, and pretreatment with PB123 protected HepG2 cells from Cd2+ toxicity measured both as cell viability (CCK8 assay) and cell injury (LDH release assay). Use of the Nrf2-inhibitor AEM1 suggested a Nrf2-dependence of the PB123-induced protection. Transcriptomic analyses of gene expression data from PB123-treated cells revealed both Nrf2-related and metallothionein-related pathways as key Reactome findings, and Detoxification as a primary Gene Ontology endpoint.

**Conclusion:**

In context with previous work by others, the present findings indicate that rosemary-based dietary supplement products like PB123 might promote beneficial responses against environmental cadmium exposure.

## Introduction

Cadmium (Cd) is a cytotoxic heavy metal and a priority pollutant that persists in the environment, therefore exposure to cadmium through food, water, or inhalation is an important health issue around the world. Notably, human exposure is likely to continue to increase due to the ongoing release of cadmium into the environment from anthropogenic sources such as mining, metallurgy, production and disposal of Ni/Cd batteries, and manufacturing and use of fertilizers, pigments, and plastics [1]. Once absorbed in the human body, cadmium is retained *in vivo* for an estimated 10 to 30 years, predominantly in the liver and kidneys [2]. The mechanism of cadmium toxicity in the liver and other organ systems is not fully determined but appears to involve oxidative stress, glutathione depletion, alterations in essential metals biology, mitochondrial disruption, and an imbalance in cellular redox status [3]. Therefore, in addition to efforts to reduce environmental exposures to cadmium and to develop and utilize chelation-based methods to reduce cadmium burden *in vivo* [4], there is interest in inducing endogenous mechanisms in the body to protect against cadmium toxicity and other heavy metals toxicity, including approaches using dietary agents [5–13].

By engaging the Antioxidant-Responsive Element (ARE) found in their promoter regions, the cellular transcription factor nuclear factor erythroid 2-related factor 2 (Nrf2) regulates the expression of many cytoprotective genes, including phase II detoxification enzymes and antioxidant enzymes. Previous publications identify a variety of antioxidant, anti-inflammatory, immune regulatory, and cytoprotective genes as being regulated by Nrf2 [14–26].

One primary system used by the body to sequester and detoxify Cd is the family of metallothionein genes and proteins, which can be regulated by the Nrf2 transcription factor. Cell exposure to Cd itself can activate the Nrf2 transcription factor [27] and increase the expression of metallothionein genes [28]. In a study of itai-itai disease in humans exposed to high cadmium levels, highly expressed metallothionein was observed in liver as a response mechanism to reduce cadmium hepatotoxic effects [29]. Both the Nrf2 pathway and metallothionein proteins play important roles in normal physiology and cell protective mechanisms. Notably there are key associations between Cd accumulation and aging [30], with fibroblasts from long-lived bird species being more resistant to cadmium toxicity [31], and metallothionein gene expression and protein levels correlating with increased life span in mammals [30,32]. Furthermore, aging is associated with decreases in the levels of Nrf2 [33–36], which could contribute to diminishment of metallothionein expression levels and its longevity-supporting benefits [30].

The aim of the present work was to examine the effects a Nrf2-activating dietary phytochemical mixture [23,37,38] on metallothionein gene expression and determine the potential benefits of phytochemical-induced Nrf2 activation against cadmium toxicity in cultured HepG2 cells.

## Materials and Methods

### Materials and reagents

PB123 extracts: rosemary extract from *Salvia rosmarinus*, ginger root extract from *Zingiber officinalis*, and luteolin from *Sophora japonica* (98% luteolin) were obtained from Jiaherb (Pine Brook, NJ, USA) and prepared in a 10:5:1 mass ratio of rosemary, ginger, and luteolin powders for PB123 as previously described [23]. Solvent extracts of PB123 mixtures were prepared overnight in ethanol at 50 mg/mL and collecting the supernatant [23,37,38]. Cell culture: antibiotics and culture media were obtained from Thermo Fisher Scientific (Waltham, MA, USA) and Lonza (Morristown, NJ). Reagents and assays: The Nrf2 inhibitor (AEM1, CAS 1030123-90-0) was obtained from Cayman Chemical (Ann Arbor, MI, USA). Cadmium sulfate (CAS 10124-36-4) and all other reagents were obtained from Sigma-Aldrich (St. Louis, MO, USA).

### Cell culture

For the genomic and cadmium toxicity experiments we used the human hepatocellular carcinoma HepG2 cell line. HepG2 cells are appropriate for the present work because they exhibit normal toxicity to cadmium exposure [9,27] and normal Nrf2 activation properties [39], lack Nrf2/KEAP1 mutations, and have previously been used in both Nrf2 activation and cadmium toxicity studies [6,8,9,40,41]. The HepG2 cells were cultured using Opti-MEM medium with 4% Fetal Bovine Serum (FBS) and penicillin/streptomycin, and were maintained by standard methods as previously described [38,42].

### Cytoprotection assays

Experiments were conducted to evaluate protective effects of PB123 pretreatment of HepG2 cells against cadmium-induced cytotoxicity, assessed by measuring changes in cell viability and changes in Lactate Dehydrogenase (LDH) release.

#### Cell viability:

Cell viability was measured using a cell counting kit-8 (CCK8) assay (Dojindo Molecular Technologies, Inc., Rockville, MD, USA) based on a water-soluble tetrazolium salt. Briefly, as previously described [23,38], cells were seeded into 96-well plates at 10,000 cells/well and cultured overnight. After the indicated treatments, CCK8 solution was added to each well according to the manufacturer’s instructions and incubated for 2 h at 37 °C. Absorbance was measured at 450 nm using a microplate spectrophotometer (Bio-Tek, Winooski, VT, USA).

#### LDH release:

LDH release was measured using an LDH assay kit assay (Cytotoxicity LDH Assay Kit-WST, Dojindo Molecular Technologies, Inc., Rockville, MD, USA). Briefly, as previously described [38], cells were seeded into 96-well plates at 10,000 cells/well and cultured overnight. After the indicated treatments, LDH levels in solution were measured according to the manufacturer’s instructions. Absorbance was measured at 490 nm using a microplate spectrophotometer (Bio-Tek, Winooski, VT, USA).

### Gene expression analysis

Briefly, HepG2 cells were treated for 16h in 24-well plates with 0 (vehicle control) or 5 μg/mL PB123 (as an extract of 50 mg/mL in 100% ethanol), with 4 biological replicates per treatment group. The cell culture and RNA isolation was performed as previously described [23]. The mRNA-seq assays and analyses were as conducted as previously reported [23,38], including processing of the raw sequencing data using Biojupies [43,44] to examine and visualize differentially-expressed genes (DEG) in PB123-treated compared to control HepG2 cells, and to examine the DEG data using Reactome pathway enrichment analysis [45]; Metascape was used for Gene Ontology analysis [46].

### Statistical analysis

Data are presented as the mean ± SEM (standard error of the mean). Significance of observed differences between treatment groups were evaluated by one-way ANOVA and Tukey’s multiple comparisons testing using Prism 9 software (GraphPad Software, version 9.4.1, San Diego, CA, USA). A *p* value < 0.05 was considered statistically significant.

## Results

### Inhibition of cadmium-induced toxicity

To investigate cadmium-induced cytotoxicity, assays measuring cell viability (CCK8 assay) and cell injury (LDH release assay) were performed using HepG2 cells. Treatment of HepG2 cells for 6h with 50 **μ**M cadmium sulfate induced a significant loss of cell viability (Figure 1A), but a 2x higher concentration of cadmium sulfate was needed to injure the HepG2 cells enough in 6h to release lactate dehydrogenase (LDH) into the culture media (Figure 1B). By both assays, pretreatment of the HepG2 cells overnight with PB123 (5 μg/mL) significantly decreased the cadmium-induced cytotoxity (Figure 1). Addition of the Nrf2 inhibitor AEM1 (5 μM) to the HepG2 cells prior to the PB123 pretreatment diminished the protective effect of PB123 against cadmium-induced loss of cell viability (Figure 1A), suggesting a role for Nrf2 activation in the protective effect.

### Differential gene expression

To investigate changes in gene expression caused by PB123 in HepG2 cells, RNA-seq data was visualized using a Volcano plot using Biojupies software [23]. PB123 treatment (5 μg/mL) induced the upregulation of 49 genes and downregulation of 21 genes with a fold change >2 and p-value <0.05. A group of metallothionein genes was notably upregulated (Figure 2). Further, the RNA-seq dataset was further examined and graphed to show the significant (P<0.001) upregulation of MT1A, MT1E, MT1F, MT1G, MT1X, and MT2A metallothionein genes (Figure 3).

### Reactome pathway enrichment analysis

Systematic pathway analysis on differentially expressed genes was performed using Reactome [45] and identified both NFE2L2-related (Nrf2) and Metallothioneins Bind Metals pathways in as three of the top four enriched pathways when comparing PB123-treated (5 μg/mL) HepG2 cells to control HepG2 cells (Figure 4).

### Gene ontology enrichment analysis

Gene Ontology (GO) enrichment analysis was performed on differentially expressed genes using Metascape tools to further elucidate biological processes that are influenced by treatment of HepG2 cells with 5 μg/mL PB123 [46]. It identified detoxification (GO:0098754) at the top of the Gene Ontology biological processes that were increased by PB123 (Figure 5).

## Discussion

In the present work, we evaluated the effects on the Nrf2-activating dietary supplement formulation PB123 on the expression of metal detoxification genes including metallothionein genes, and on the inhibition of cadmium-induced hepatic cytotoxicity in cultured HepG2 cells. Pretreatment of HepG2 cells with an extract of PB123 (5 μg/mL) attenuated the cytotoxic effects of cadmium sulfate (50 μM), measured as cell viability using CCK8 assay. Furthermore, inhibition of Nrf2 during the PB123 pretreatment (using Nrf2 inhibitor AEM1, 5 μM) diminished the beneficial effects of the PB123 pretreatment on cell viability, suggesting Nrf2-dependence of the protection by PB123; since the AEM1 Nrf2 inhibitor can also have unwanted antiproliferative side effects, future work will include more specific approaches to Nrf2 inhibition. Likewise, PB123 pretreatment of HepG2 cells (5 μg/mL) attenuated the cytotoxic effects of cadmium sulfate (50 μM) when measured by LDH release assay. Analysis of differentially expressed genes in HepG2 cells treated with PB123 (5 μg/mL) compared to untreated HepG2 cells identified Nrf2, metallothionein, and detoxification as top Reactome and GO pathways upregulated by the treatment, and examination of the RNA-seq data revealed significant upregulation of the MT1A, MT1E, MT1F, MT1G, MT1X, and MT2A metallothionein genes by PB123.

This work is supportive of previous findings that rosemary-based dietary supplement products might promote healthful responses to environmental cadmium challenges including work by Sakr, et al., in which a rosemary extract fed orally to rats was protective against cadmium-induced liver damage, and work by Das, et al., in which the rosemary compound carnosic acid attenuated cadmium-induced cytotoxicity in cultured kidney cells *in vitro* and protected mouse kidney function *in vivo* [10,47].

Khan, et al., found that caffeine protected against cadmium-induced neurotoxicity by a mechanism including Nrf2 regulation [48]. In a recent review article, Ashrafizadeh, et al., concluded with the possibility that Nrf2 activators could be utilized to decrease cadmium-induced toxicity [49], in which they stated: “…targeting Nrf2 signaling pathway and, subsequently, improving antioxidant balance can be considered as a potential candidate in combating with cadmium toxicity. At the present review, we describe how the Nrf2 signaling pathway can be modulated to decrease cadmium toxicity.” In the present work, we build on this concept and report that our findings using the PB123 Nrf2-activating dietary supplement formulation offers further support for it.

Because the Nrf2 transcription factor is known to play a role in both metallothionein gene regulation and cellular protection against cadmium toxicity, and since metallothionein gene products are known to contribute to cellular protection against heavy metals such as cadmium [50], the linkage between these Nrf2 effects in the present work is not unexpected. Furthermore, cadmium itself can induce cellular upregulation of metallothionein genes [51], and prior work with dietary phytochemicals like naringin from citrus have shown protection against cadmium-induced liver cell toxicity [9]. The limitations of the present study include that it was conducted on cultured cells *in vitro* with acutely toxic doses of cadmium, so does not address potential *in vivo* effects against chronic low levels of cadmium. Also, the transcriptomic data established a correlation between upregulation of MT1 genes and the beneficial actions of PB123 against cadmium toxicity, but further study should be conducted on MT proteins and their activity.

## Conclusion

In the present work we identify a practical, actionable approach to increasing cytoprotection against cadmium toxicity using a Nrf2-activating phytochemical approach in cultured HepG2 cells. The PB123 formulation is a Nrf2-activating dietary supplement mixture [23,38,42,52,53], and in the present work PB123 demonstrated genomic upregulation of metal binding and detoxification pathways, potent metallothionein gene transcription upregulation, and increased protection against cadmium-induced cytotoxicity in cultured HepG2 cells. The results indicate that dietary Nrf2 activators, especially those containing carnosol and carnosic acid from rosemary [47], merit further study and consideration for use in dietary approaches to support endogenous cytoprotective mechanisms against the common environmental pollutant cadmium.

## Figures and Tables

**Figure 1:**
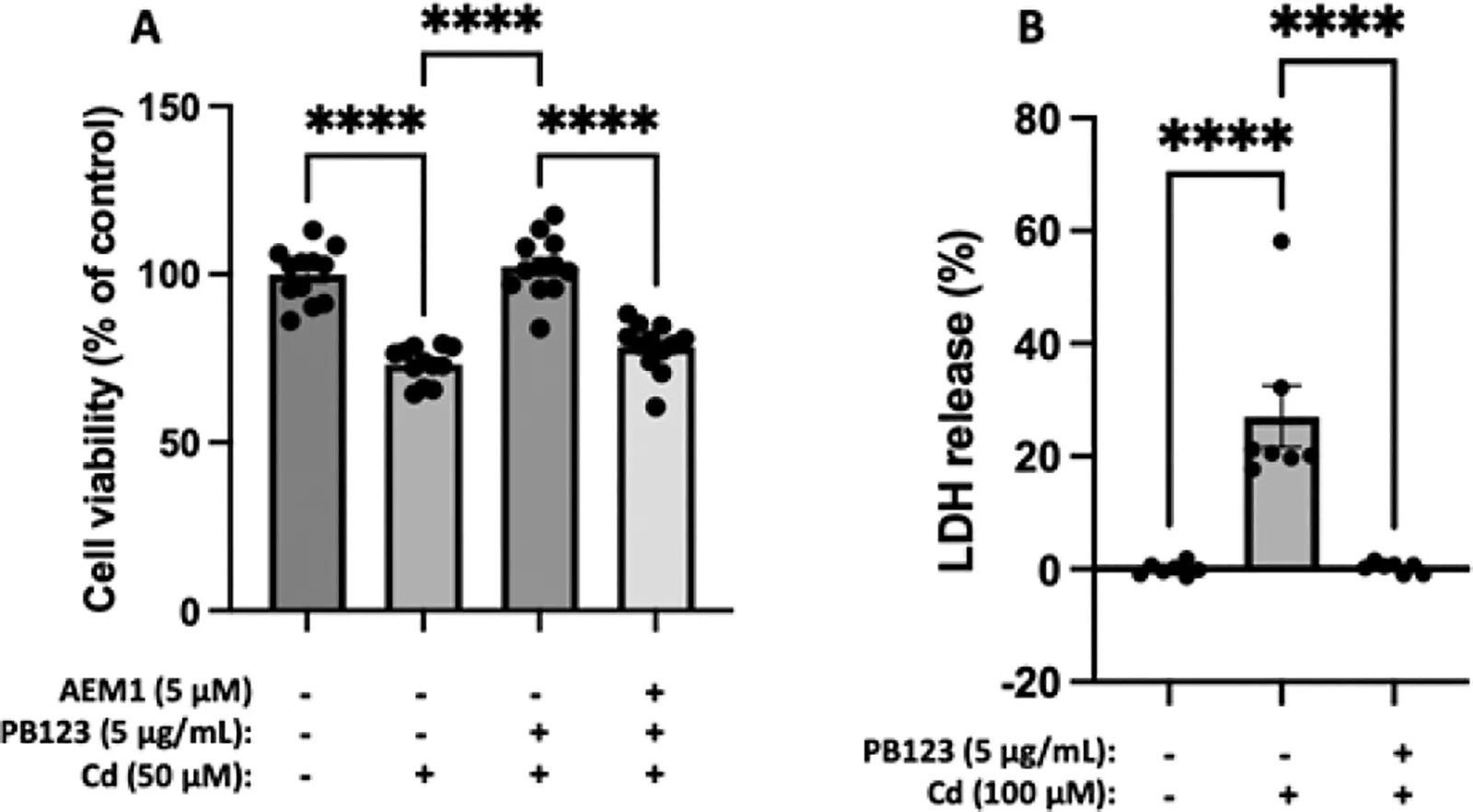
Pretreatment (24h) of HepG2 cells with PB123 (5 μg/mL) decreased cadmium-induced (50 μM) loss of cell viability, and the protective effect was blocked by cotreatment with the AEM1 Nrf2 inhibitor (5 μM) (A), and also decreased cadmium-induced cell injury (measured as % LDH release) following 6h of treatment with cadmium sulfate (100 μM). (****p<0.0001).

**Figure 2:**
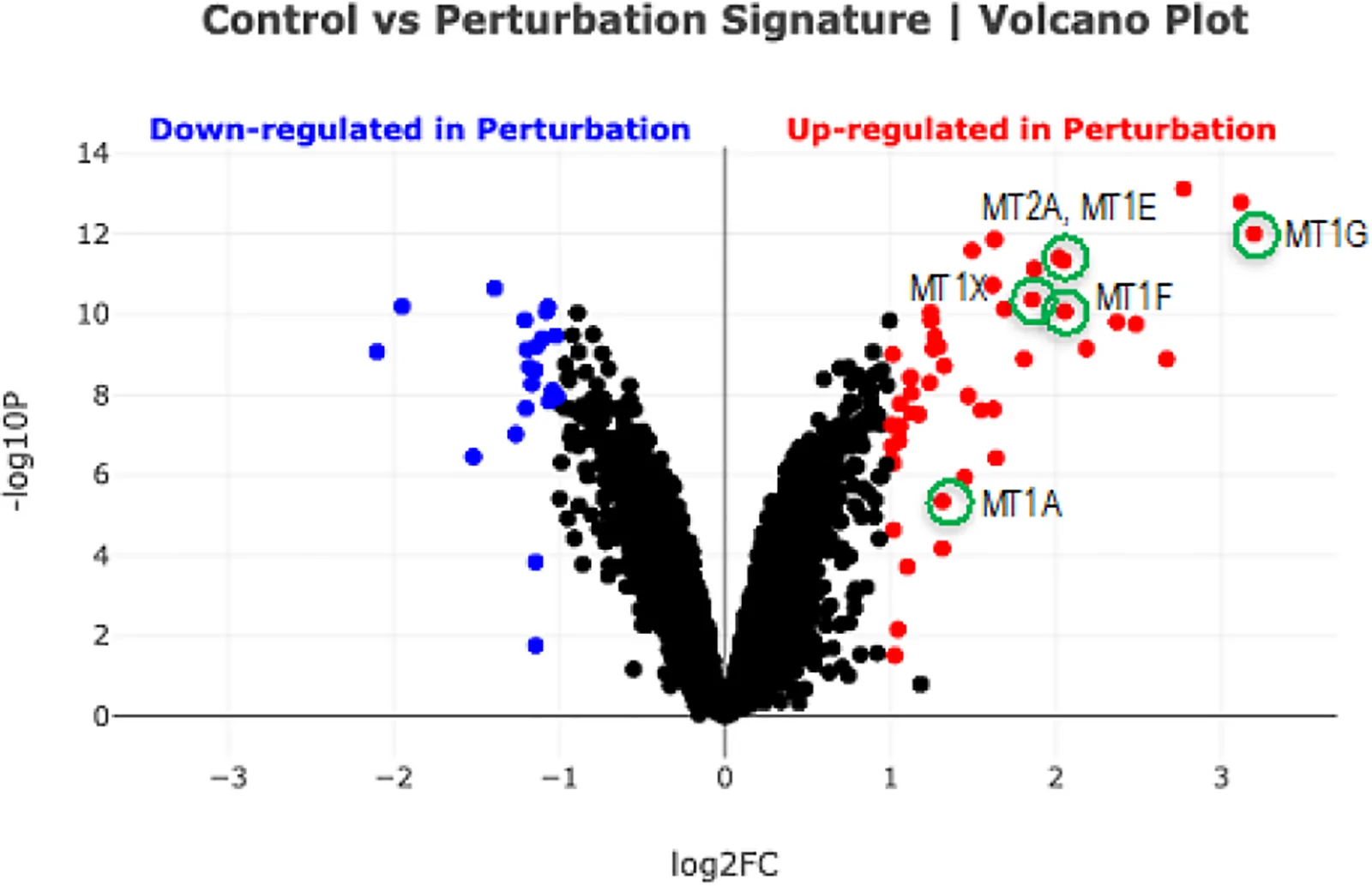
BioJupies was used to generate a Volcano plot of differentially expressed genes that were either up or downregulated in HepG2 cells treated with 5 μg/mL PB123 compared with vehicle-treated control HepG2 cells. Red indicates upregulated genes and blue indicates downregulated genes. The metallothionein genes (circled in green) are labeled.

**Figure 3:**
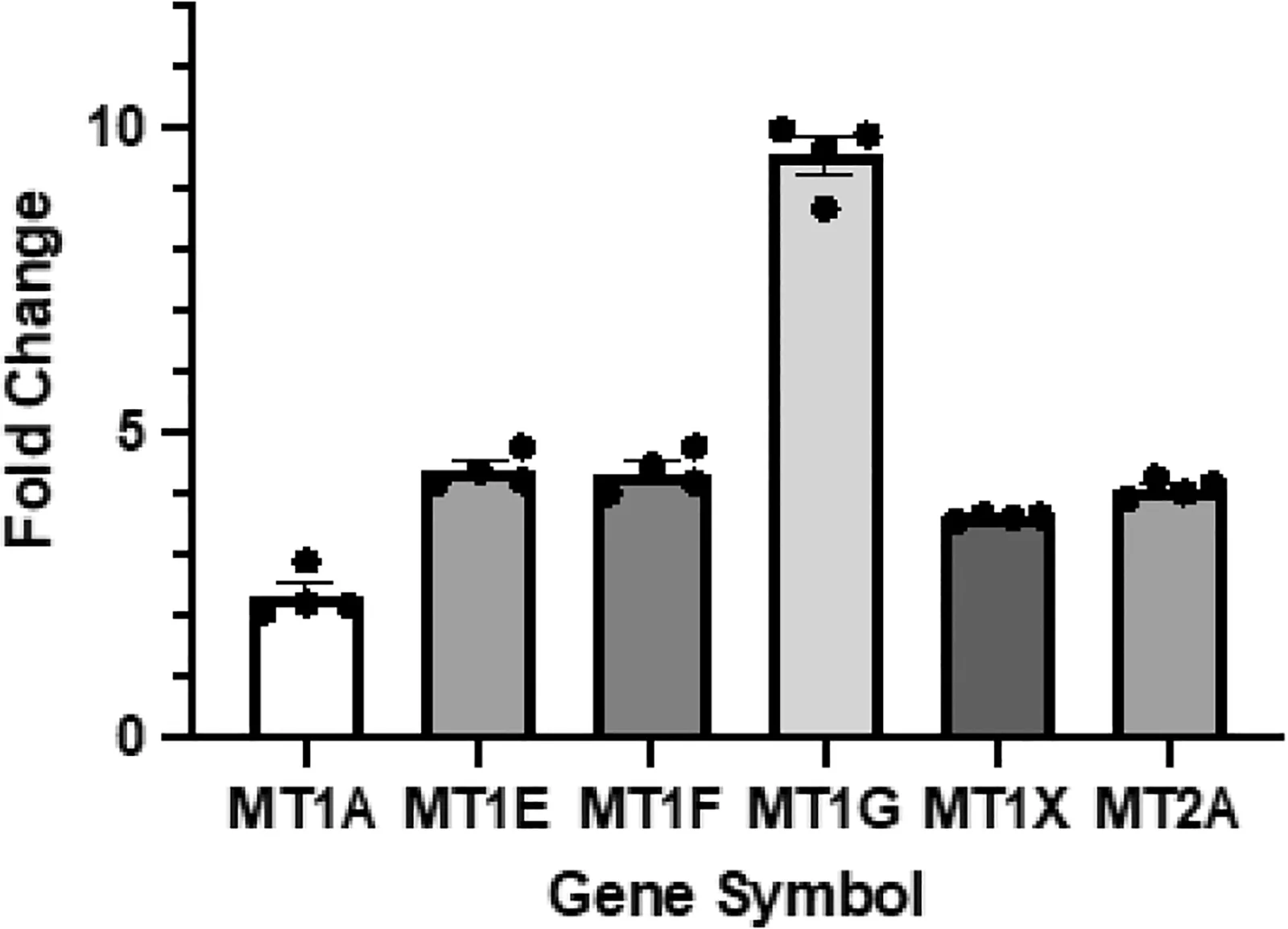
HepG2 RNA-seq 5 μg/mL PB123, p<0.001 for differential expression of each of the genes shown in PB123-treated HepG2 cells versus untreated HepG2 cells.

**Figure 4:**
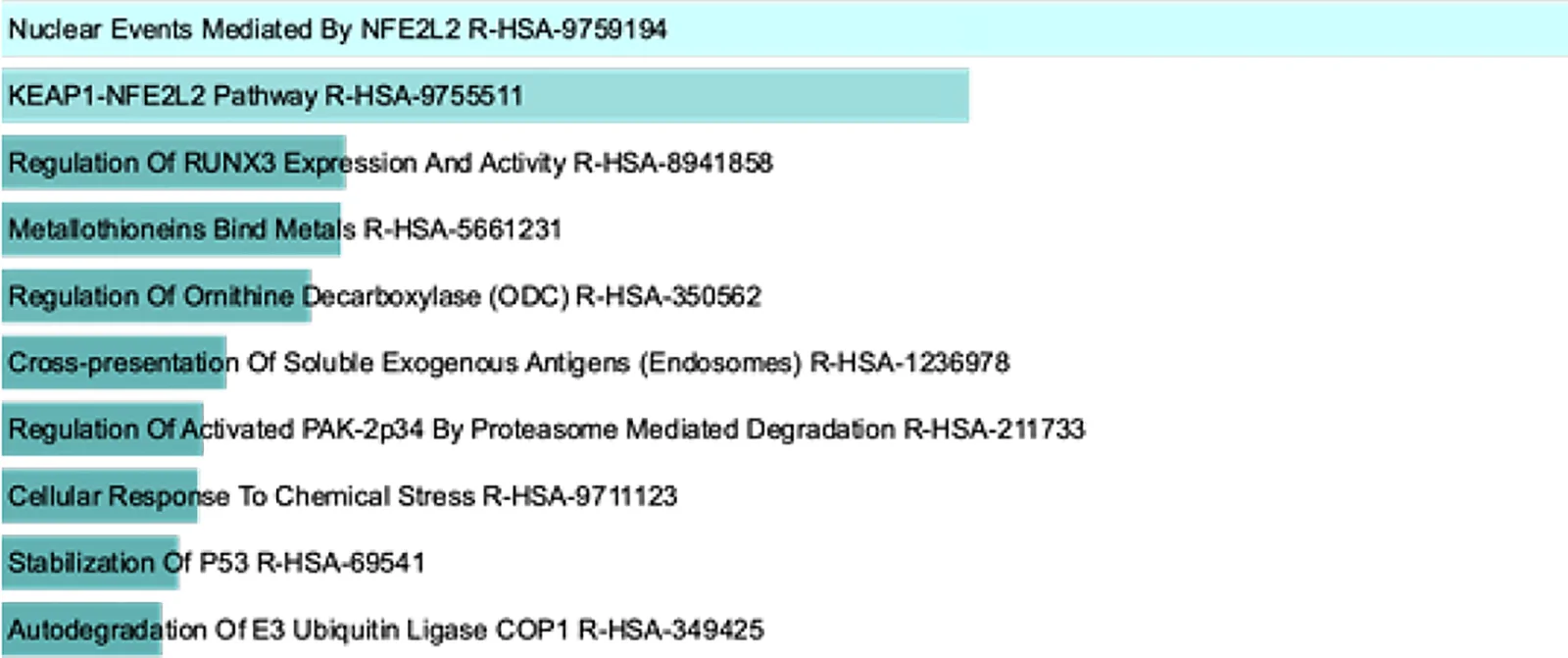
Reactome pathway enrichment analysis of the differentially expressed genes in 5 μg/mL PB123 treated vs. control treated HepG2 cells identified several pathways relevant to metals detoxification, including Nuclear Events Mediated By NFE2L2 R-HSA-9759194, KEAP1-NFE2L2 Pathway R-HSA-9755511, and Metallothioneins Bind Metals R-HSA-5661231.

**Figure 5:**
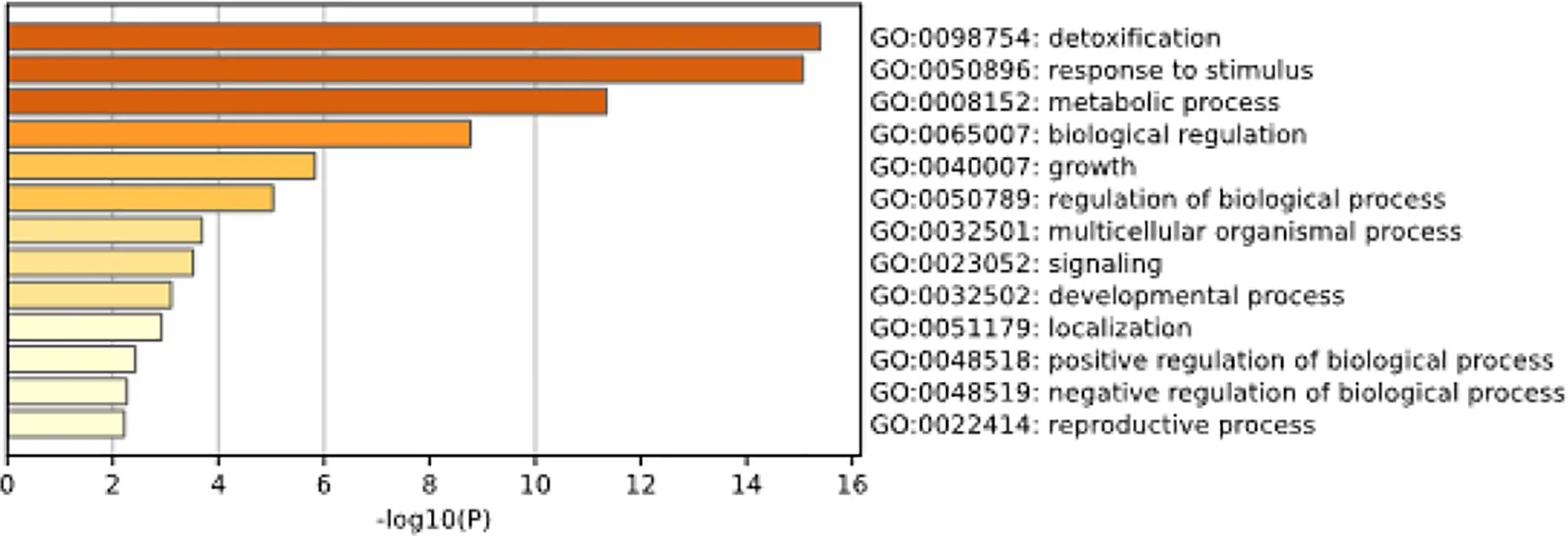
Gene Ontology (GO) pathway enrichment analysis was obtained using Metascape on the top 100 differentially expressed genes in 5 μg/mL PB123 treated vs. control treated HepG2 cells, and it identified GO:0098754: detoxification in the top-level Gene Ontology biological processes.

## Data Availability

All relevant data is contained within the manuscript.

## Abbreviations

ARE: antioxidant response element
GO: gene ontology
LDH: lactate dehydrogenase
MT: metallothionein
Nrf2: nuclear factor erythroid 2-related factor 2
